# The Institutional Worker

**Published:** 1913-06-07

**Authors:** 


### INSTITUTIONAL WORKER

**Being a Special Supplement to "The Hospital"**

### OUR BUREAU OF INFORMATION.

### Rules for Correspondents.

1. Every letter must be accompanied by the coupon to be cut from the back cover (inside page) of THE HOSPITAL, current issue, and must contain the name and address of the correspondent with pseudonym for publication if desired. Replies by post cannot be given, save under exceptional circumstances at the Editor's discretion.

2. Letters from Approved Homes in reply to special needs published in the Bureau should state terms and full particulars, and be sent prepaid under cover to the Editor of the Bureau with name written across coupon for identification.

3. Proprietors of Homes which have not yet been entered on the List of Approved Homes, but have spare accommodation likely to suit special needs, are invited to write for an application form for registration. The fee for registration, which includes two announcements of the Home in the Bureau and other privileges, is 10s.

4. All communications to be addressed to the Editor of THE HOSPITAL, 28 Southampton Street, Strand, London, W.C., and marked "Bureau of Information."

### *THE SICK AND DISTRESSED.*

**The Editor is prepared to make known without charge the needs of any who may be sick or in difficulties, and to guide them in making choice of Homes for treatment or convalescence. Reduced terms can often be arranged with Homes on the Approved List for those unable to pay full fees. Queries are specially invited from those who are engaged in any kind of philanthropical work. A new edition of the leaflet describing the purposes of the Bureau of Information is now ready and will be sent free of charge on application.**

### Choice of Homes.

THE opportunity afforded in these columns for bringing the varied classes of invalids into touch with small nursing homes, able to give them the accommodation and treatment they require, should be more fully appreciated than it is. The need for such homes, where various chronic, medical, surgical, maternity and other cases can be received at a moderate charge, is much felt, and though there are a number of small homes, the choice of one suitable for the class of ailment to be treated, the management of which can be relied upon, is often a difficult matter. These columns offer the opportunity to those in need of assistance in this respect to make known their wants free of charge under a pseudonym; and through this medium they are placed in communication with the homes able to give the particular accommodation required.

Our system of registration overcomes the difficulty, with which those seeking such accommodation are faced, of discriminating between well-managed and undesirable homes, for no home is added to our register until we are satisfied by direct medical and other testimony as to its suitability for dealing with the class of cases received, and as to its general good management.

### Help for Phthisical Case Required.

WE are desirous of finding for a young woman suffering from advanced phthisis a suitable Home or the means whereby she may be properly attended to. Several efforts have been made to get her into a sanatorium, but her condition is regarded as too hopeless. As she has been deserted by her husband and her friends are very poor, no funds are available for her maintenance. Such assistance as any of our readers can render will be gratefully welcomed. Replies should be addressed "H. E. B., 160B."

### *THE INSTITUTIONAL ARTIFICER.*

**QUESTIONS FOR JUNE.**

**1. Describe and, if possible, give a sketch or photograph of any home-made piece of hospital furniture or apparatus you have designed, or which you have found to serve a useful purpose at your institution.**

**2. Describe any defects you have found in your heating and hot-water system, and the steps you have taken to remedy them.**

*NOTE.—A minimum payment of FIVE SHILLINGS will be made for each descriptive note based upon the foregoing questions which is accepted for publication.*

RULES.

The following rules must be observed:—

1. Contributions must be written on one side of the paper. They must bear the name and address of the sender and be accompanied by coupon to be cut from the back cover (inside page) of the current issue of THE HOSPITAL. A pseudonym must be chosen if the name is not to be published.

2. They must be addressed to the Editor of THE HOSPITAL, 28 & 29 Southampton Street, Strand, London, W.C., so as to reach him before the end of the month, and must be marked in the left-hand corner "Institutional Artificer."

### Apparatus for Fowler's Position.

NOT a few hospitals lack the apparatus necessary for placing their patients in *Fowler's Position*, and in the absence of it various attempts are made to secure the position by placing the legs of the bedstead on chairs or lockers, with the result that whilst the desired object is attained, these articles of furniture are badly damaged, and the bedsteads strained. There are several excellent attachments, specially designed for this purpose by the bedstead manufacturers, but their cost possibly precludes their more general use. A special pillow and straps, designed by Mr. Whiteford, of Plymouth, and manufactured by Messrs. Allen and Hanburys, a full description of which appeared in THE HOSPITAL of May 24, offers a solution of the difficulty, for experience seems to indicate that it answers admirably; it may be obtained at a moderate price.

### Swivel Baths.

THE inconvenience of a small bath room may be overcome to a large extent by adopting a plan in use at the Western Infirmary, Glasgow; here will be seen a bath designed to swing on a pivot. When the bath is not in use it can be placed against the wall. When required for use it can be so arranged that it is possible for the nurse or attendant to approach it from either side.—"S. W."

### *THE INSTITUTIONAL OFFICER.*

Contributions from officers engaged in the work of institutions for the relief of the sick and afflicted, whether rate-supported or otherwise, are invited, and will be paid for in accordance with scale if made use of.

### Voluntary and Poor-Law Officers.

"INSTITUTIONAL WORKER" writes: The need for co-operation between the many agencies dealing with the sick and needy is so great that it seems surprising that the relations between the voluntary hospital workers and the Poor-Law officers are not more harmonious than they are. More often than not, instead of co-operation one meets with apparent bad feeling between these two bodies; yet an analysis of the grievances would probably reveal no substantial basis for them. The truth is that the voluntary workers do not make themselves thoroughly acquainted with the functions of the Poor-Law authorities, and possibly the latter do not sufficiently recognise the extent to which the operations of the voluntary hospitals relieve the poor rate.

### The Advantage of Mutual Service.

My own experience is that the Poor-Law officers can be extremely helpful to the hospital workers, and on many occasions I have been indebted to them for much valuable information and assistance. Then, too, there are cases of patients discharged from the hospitals who may be in need of some special assistance, such as the provision of an artificial limb, the full cost of which cannot readily be found; if the notice of the Guardians be drawn to such cases, they often find it possible to contribute the needful help, especially when such help will be the means of making the patient independent of the rates. On the other hand, there are many ways in which the hospitals can serve the infirmary or workhouse inmate by giving special advice or treatment.

The fact that the Poor-Law Guardians have power to, and do, subscribe to numerous voluntary agencies in order to secure such special aid as the needs of the afflicted coming within their jurisdiction may indicate, is sufficient evidence of the possibilities for good which earnest co-operation would bring about, and an exchange of experiences by the officers representing the two bodies in question and the discussion of their functions and difficulties is much to be desired.

### Visitors to Children's Wards.

"An Observer" writes: The prohibition by some hospital authorities of visitors to the children's ward is not infrequently the cause of much discontent amongst the parents as well as genuine distress. The arguments against the visitation of children by their parents is that their progress is interrupted by the unsettling influence which a visit often creates, and, further, that there is the danger of introducing infection to which children are more susceptible than adults. There is, no doubt, a great deal to be said in favour of these arguments; but, on the other hand, the rule is stultified by the frequent exceptions which are made to it in order to keep the peace with importunate parents. The rule seems to be inhuman, and it demands a good deal of faith in the hospital on the part of the parent who, after all, has only the word of the hospital authority, too often conveyed, in a perfunctory manner, by a subordinate, as to the welfare of the child. The abolition of such a rule, and the exercise of discretion in the admission of visitors, seems to be a reasonable alternative.

---

### Grouping of Out-patients.

The difficulty you experience in dividing the patients into their proper groups while awaiting attention by the medical officers to whom they have been allocated can be quite easily overcome by a little method in the arrangement of seats. You will find it best to place these in groups opposite the consulting rooms, and a finger-post, attached to each group, bearing the name of the physician or surgeon in attendance, will obviate any risk of confusion.—"Hospital Clerk."

### *INSTITUTIONAL FACTS AND FIGURES.*

**QUESTION FOR MAY.**

**During a period of four weeks in the winter months state what was the quantity of gas consumed in your institution for purposes other than illuminating, its approximate cost, the quantity of each kind of fuel used and its cost, deducting the cost of fuel for laundry purposes (if any).**

**In answering this question the following particulars should be given: (1) Number of wards and size of each (in beds); (2) total number of coal fires (a) in the wards, (b) in the rest of the building; (3) the number of gas-cooking and other stoves and sterilisers; (4) the name of the heating and hot-water supply service.**

"Institutional" sends the following reply:—Consumption of gas, 262,100 cubic feet, costing £22 10s. 6d. (of this 102,600 cubic feet was consumed in our kitchen department). Consumption of other kinds of fuel and its cost: (1) 252 tons boiler coal, costing £179 11s.; (2) 40 tons house coal costing £20. (1) We have sixteen wards averaging twenty-seven beds each. (2) Our total number of coal fires is (a) in the wards, 130; (b) in the rest of the building, 160. (3) We have twenty-one ordinary gas-cooking stoves (sixteen of which are in ward kitchens), twelve gas rings, thirty-two gas jets, three gas sterilisers (many of our sterilisers are steam), two gas grills and toasters; these are in addition to our gas roasters and cooking appliances in our kitchen department. (4) We have the ordinary low-pressure heating system, heated by Rolyes' patent calorifiers.

---

### *EMPLOYMENT AND TRAINING.*

### Nursing in Rhodesia and South Africa.

Rhodesia.

We have no information of nursing co-operations in Rhodesia. The British South Africa Company has hospitals in many towns, the largest being at Salisbury. At Bulawayo there is a public Memorial Hospital, supported partly by the Administration and partly by voluntary contributions. The Rhodesia Committee of the South African Colonisation Society has a Maternity and Children's Nursing Home at Salisbury, with a lady superintendent and staff of trained nurses.

South Africa.

There is a Nurses' Co-operation Institute in Cape Town and another in Kimberley. The fees vary from £3 3s. to £4 4s. per week, from which 7½ per cent. is deducted. A moderate charge is made for food and the use of a bedroom when not at a case. The South African Colonisation Society gives to approved candidates, going to definite posts, 15 per cent. reduction on second-class accommodation by the Union-Castle Line, and arranges for them to be met at the port of landing and escorted to their destinations. For posts at Surgical Nursing Homes, a three years' general training at a recognised training school is necessary; for midwifery posts, the C.M.B. certificate is required in addition. If you desire to take advantage of the facilities offered by the South African Colonisation Society, apply to the Secretary, 23 Army and Navy Mansions, Victoria Street, London, S.W.—"Bradford."

### Midwife and Masseuse.

A coupon should be enclosed with each inquiry. We cannot give postal replies. The only way to obtain a position such as you require is to study the advertisements which appear in the Nursing Journals and reply to them.—"G. K."

### Mental and Monthly Nursing.

A coupon should be enclosed with each inquiry. We cannot give postal replies. You do not say whether you require work as a mental or monthly nurse, in which special branches you hold certificates. The absence of a general training will make it difficult, if not impossible, for you to obtain a post in a London nursing institution. Your best course would be to study the advertisements which appear in the Nursing Journals and reply to them. As regards maternity work, the Rural Midwives' Association, 47 Victoria Street, S.W., could help you.—"A. R."

### Electricity and Massage.

A coupon should be enclosed with each inquiry. We cannot give postal replies. We do not think it likely that you will, in return for your services as a masseuse, obtain at any institution a course of training in electrical treatment in addition to a salary. The Incorporated Society of Trained Masseuses, 12 Buckingham Street, Strand, W.C., acts as a registry for its members, and could possibly render you some assistance.—"K. W. H."

---

### *EDITOR'S LETTER-BOX.*

"Kindness."—We should be interested to hear whether, as a result of our suggestion, you have been successful in obtaining a suitable Home for the lady's maid in whom you are interested.

"H. E. B."—We have not so far received any response to the appeal we published on behalf of the young woman suffering from advanced phthisis in whom you are interested, and we are therefore drawing further attention to your need this week. We should be interested to know whether you made application to the institution suggested, and with what result.

"J. T. S."—Can we be of any further assistance to you, or have you been successful in obtaining a suitable home for the case in which you are interested? We forwarded you a communication from one of our readers some time ago, and we should be glad to hear whether you were able to make a satisfactory arrangement.

---

The Editor will be glad to receive correspondence and to consider contributions upon all subjects relating to institutional work, and affecting the welfare of institutional officers.

### Editor's Notices.

**Contributions:**

Contributions should be written, or preferably typed, on one side of the paper only, and all articles sent in are accepted upon the distinct understanding that they are forwarded to THE HOSPITAL only.

The Editor cannot undertake to return MSS. not used, but when a stamped directed envelope is enclosed the MSS. may be returned if a special request is made.

Accepted articles and paragraphs of news will be paid for after publication at the scale rate.

**Address:**

To prevent delay all contributions and letters on editorial business must be addressed exclusively to the Editor, "The Hospital," 29 Southampton Street, Strand, London, W.C. It is important that this regulation shall be strictly observed.

**Correspondence:**

Correspondence on all subjects is invited. The name and address of correspondents must be given as a guarantee of good faith, but not necessarily for publication.

**Special Articles:**

Special articles are invited, and questions, inquiries, and paragraphs upon all matters relating to the work, administration, and management of general, special, mental, fever, cottage and convalescent hospitals, sanatoria, homes, institutions, societies and organisations for the treatment or care of the sick, injured and dependants of all classes. Every matter affecting the interests and welfare of the staff of all grades working in these institutions will receive special consideration.

**Administrative Medicine, Research and Items of News:**

Special terms will be paid for approved articles on subjects in this wide field by experts who have made some section of it their special study and interest. We wish to give prominence to every movement tending to advance accuracy of diagnosis, efficiency in treatment, and the development of modern methods to eradicate disease and promote the welfare of the suffering.

**A Bureau of Information:**

We invite inquiries and applications for information and help in providing for that numerous class of sufferers who require special care or house-room which they cannot obtain in their own homes or secure for themselves. **We cannot, however, prescribe, or recommend practitioners.**

**Books for Review:**

Publishers are particularly requested to send advance proofs of any new books of importance whenever possible, as the Editor has made arrangements to publish immediate reviews on a new plan.

**Photographs, Plans, Blocks, and Illustrations:**

It is requested that wherever possible MSS. may be accompanied by illustrations in any of the above forms. The name of the sender and of the article to which it belongs should be written on the back of each photograph, drawing, or block for purposes of identification.

**Local Papers:**

Newspapers containing reports or news paragraphs should be marked and addressed to the Sub-Editor.

**The Coupon System:**

A coupon will be found at the bottom of the third inside page of the cover each week. This coupon must be attached to every question or inquiry to which an answer is desired in THE HOSPITAL.

### Our Advertisement Pages.

NOT the least of the many interesting and unique features of this week's issue of THE HOSPITAL is the useful information contained in the advertisement pages. Here will be found the results of commercial enterprise in its efforts to secure the most efficient appliances for the economical management of every department of institutional work. Consequently, the particulars contained in these pages cannot fail to prove of the utmost assistance to every keen and progressive institutional official. It is our purpose to make the pages of THE HOSPITAL as interesting and as comprehensive as possible, so that it is *the journal* of all that is essential and up to date in hospital and institutional affairs. In these circumstances, we would submit that our readers will find it to their interest to give some time and attention to our advertisement pages.

### Manager's Notices.

**Letters relating to the publication, sale, and advertising departments of "The Hospital" must be addressed to The Manager, "The Hospital," The Hospital Building, 28 and 29 Southampton Street, London, W.C.**

**Subscriptions** may begin at any time, and are payable in advance. Cheques and Post-Office Orders should be crossed London County and Westminster Bank, Covent Garden Branch, and made payable to the Manager of THE HOSPITAL.

RATES OF SUBSCRIPTION (payable in advance).

UNITED KINGDOM.

| | | |
|---|---|---|
| Three Months (including Postage) | | 2s. 0d. |
| Six Months | do. | 4s. 0d. |
| Twelve Months | do. | 6s. 6d. |

FOREIGN AND COLONIAL.

| | | |
|---|---|---|
| Three Months (including Postage) | | 2s. 6d. |
| Six Months | do. | 5s. 0d. |
| Twelve Months | do. | 8s. 8d. |

**Advertisements:**

To ensure insertion, all advertisements for THE HOSPITAL must reach the Manager not later than Wednesday morning in each week. For scale of charges see pages vii and 4.

**Special Rates** will be quoted for those institutions that agree to send all their vacancy, contract, and public notices to THE HOSPITAL each year, so as to make it a file of such announcements for ready reference.

This form may be used for small prepaid advertisements, and when filled up should be posted to

*The Manager*, THE HOSPITAL,
28 and 29 Southampton Street,
Strand, W.C.

Appointments Wanted, 21 words ... 1s. 0d.
Appointments Vacant, 21 words ... 2s. 6d.

### HANDBOOKS
### For TRAINED WORKERS

POCKET-GUIDE SP SERIES

1/- net. 1/1 Post free.

**Bound in Limp Cloth.**
**Suitable Size for the Pocket.**

The works which comprise the S.P. Pocket Guide Series are intended for ready reference, consequently the aim of the Publishers has been to make them as concise and lucid as possible. Each book is written by an expert on the subject of which it treats, and the utmost reliance therefore can be placed in the information it imparts.

**Essentials of Fever Nursing.** By LYTTON MAITLAND, M.D. (Lond.), M.B., B.S., D.P.H. (Camb.).

**Manual and Atlas of Swedish Exercises.** (*With over 60 Illustrations.*) By THOMAS D. LUKE, M.D., F.R.C.S.

**Bandaging Made Easy.** (*Illustrated with over 90 Diagrams.*) By M. HOSKING, Sister-in-Charge, Tredegar House, Bow, E.

**How to Write and Read Prescriptions.** By LYTTON MAITLAND, M.D. (Lond.), M.B., B.S., D.P.H. (Camb.).

**Principal Drugs and their Uses.** By A PHARMACIST.

**Asepsis and How to Secure It.** By H. W. CARSON, F.R.C.S.

**Treatment after Operations.** *Rules for Nursing after General and Special Operations.* By MARY WILES.

**The Nurse's Duties before and during Operations.** By MARGARET FOX, Matron, Prince of Wales's General Hospital, Tottenham.

**Other works, in amplification of this Series, will be announced in due course.**

*Published by*

**The SCIENTIFIC PRESS, Ltd.**

**28/29 Southampton Street, STRAND, LONDON, W.C.**

